# Metabolic reprogramming in *Helicobacter pylori* infection: from mechanisms to therapeutics

**DOI:** 10.3389/fcimb.2025.1678044

**Published:** 2025-10-13

**Authors:** Tong Liu, Xuelin Zhao, Ting Cai, Wei Li, Minglin Zhang

**Affiliations:** ^1^ Department of General Surgery, Zhongshan Hospital of Traditional Chinese Medicine Affiliated to Guangzhou University of Traditional Chinese Medicine, Zhongshan, Guangdong, China; ^2^ Department of Gastroenterology, The Third Xiangya Hospital, Central South University, Changsha, Hunan, China; ^3^ Department of Gastroenterology, Hunan Provincial People’s Hospital, the First Affiliated Hospital of Hunan Normal University, Changsha, Hunan, China; ^4^ Hunan Provincial University Key Laboratory of the Fundamental and Clinical Research on Functional Nucleic Acid, Changsha Medical University, Changsha, Hunan, China

**Keywords:** *Helicobacter pylori*, metabolic reprogramming, lipid metabolism, glucose metabolism, lactate metabolism, amino acid metabolism, gastric carcinogenesis

## Abstract

*Helicobacter pylori* (*H. pylori*), a key gastric mucosal pathogen, causes chronic gastritis, peptic ulcers, and gastric cancer. *H. pylori* remodel the gastric microenvironment through metabolic reprogramming to drive pathogenesis. CagA^+^ strains disrupt lipid metabolism, increasing non-alcoholic fatty liver disease, cardiovascular, and Alzheimer’s risks via PPAR interference, *GBA1* demethylation, and altered FABP1/APOA1 expression, reversible by eradication. In glucose metabolism, *H. pylori* promote carcinogenesis via Lonp1-induced glycolysis, PDK1/Akt dysregulation, and HKDC1/TGF-β1/MDFI-mediated epithelial-mesenchymal transition, while exacerbating high-fat diet-induced dysbiosis. Infection manipulates macrophage immunometabolism. Bacterial utilization of host L-lactate through *H. pylori* gene clusters enables proliferation, gland colonization, and immune evasion by suppressing complement activation and TNF/IL-6 secretion. Lactate-targeting strategies show therapeutic promise. Amino acid dysregulation involves *H. pylori* biotin protein ligase (HpBPL)-mediated catabolism and γ-glutamyl transpeptidase-induced glutathione hydrolysis, depleting antioxidants while inducing dendritic cell tolerance. branched-chain amino acids accumulation activates mTORC1, and cystine-glutamate transporter inhibition with miR-30b upregulation exacerbates mucosal damage, forming a self-sustaining “metabolic reprogramming-immune evasion-tissue destruction” cycle. These mechanisms collectively enable *H. pylori* to propel gastric carcinogenesis, highlighting metabolism-targeted interventions as future solutions. This review summarizes how *H. pylori* remodel the gastric microenvironment and drives pathogenesis by manipulating host lipid, glucose, lactate, and amino acid metabolism.

## 1 Introduction

*Helicobacter pylori* (*H. pylori*) is a Gram-negative, microaerophilic, spiral-shaped bacterium that colonizes the gastric mucosa and damages gastric epithelial cells (GECs). In 1982, Warren and Marshall first discovered this bacterium, identified it as a cause of chronic gastritis, and successfully isolated it. Their groundbreaking work was later awarded the Nobel Prize (Warren and Marshall, 1983; Pincock, 2005). *H. pylori* is the most common cause of chronic gastritis and can lead to peptic ulcers, gastric cancer (GC), and gastric mucosa-associated lymphoid tissue (MALT) lymphoma in some patients (Arnold et al., 2020; Malfertheiner et al., 2023; Salvatori et al., 2023). The diverse pathological changes induced by *H. pylori* infection arise from interactions between the host’s genetic makeup, environmental factors, and bacterial virulence factors, resulting in different phenotypes of chronic gastritis: antral-predominant, corpus-predominant, or pangastritis (Baj et al., 2020; Sharndama and Mba, 2022; Tran et al., 2024; Duan et al., 2025). Globally, approximately 50% of the population is infected. While about 80% of carriers are asymptomatic, all infected individuals develop gastritis. Significant geographical variations exist in prevalence, though a general declining trend is observed. Classified as a Group 1 carcinogen by the World Health Organization, *H. pylori* is a major risk factor for GC (Che et al., 2022; Li et al., 2023; Chen et al., 2024). Its ability to robustly survive in the highly acidic gastric environment and evade host immune responses makes it a key driver in the progression of gastrointestinal diseases.

The pathogenesis of *H. pylori* is increasingly linked to its ability to manipulate host cellular processes, particularly metabolic reprogramming. This process refers to the adaptive alteration of metabolic pathways, fluxes, and key metabolite levels in response to environmental changes, playing a pivotal role in cancer malignant transformation, immune cell activation, and pathogen infections. Within the tumor microenvironment (TME), tumor cells reprogram glucose, lipid, and amino acid metabolism to competitively deplete critical nutrients (e.g., glucose, glutamine, arginine) and accumulate inhibitory metabolites (e.g., lactate), thereby suppressing CD8^+^ T cell function and proliferation to facilitate immune evasion (Tharp et al., 2024; Liang et al., 2025). The metabolic reprogramming of immune cells further fosters an immunosuppressive TME that dampens antitumor immunity. Targeting these metabolic pathways (e.g., combining immune checkpoint blockade (ICB) with metabolic inhibitors) and exploring the role of gastrointestinal microbiota in the TME represent potential therapeutic strategies (Zhao et al., 2022; Lin et al., 2024; Zhang et al., 2024).

In gastric disease research, metabolic reprogramming features prominently. For instance, adipocytes promote GC omental metastasis via phosphatidylinositol transfer protein, cytoplasmic 1 (PITPNC1)-mediated fatty acid metabolic reprogramming (Tan et al., 2018). Fenofibrate reprograms glycolipid metabolism in GC cells by acting on carnitine palmitoyl transferase 1 (CPT1) and fatty acid oxidation pathways, activating the AMPK pathway, and inhibiting the HK2 pathway, thereby suppressing proliferation and inducing apoptosis (Chen et al., 2020). LIM Homeobox 9 (LHX9), highly expressed in GC, transcriptionally activates pyruvate kinase M2 (PKM2) to induce glycolytic reprogramming, enhancing the malignant properties of GC stem cells and driving tumor progression (Zhao et al., 2023). Beyond tumor cells themselves, pathogen infections also involve metabolic reprogramming: *H. pylori* develops drug resistance and undergoes virulence-associated metabolic alterations when exposed to clarithromycin *in vitro* (Rosli et al., 2024). Under cytotoxin-associated gene A^+^ (CagA^+^) *H. pylori* infection, mitochondrial Sirtuin 3 (SIRT3) deficiency promotes reactive oxygen species (ROS) generation by inhibiting its deacetylase activity, thereby stabilizing HIF-1α protein and enhancing its transcriptional activity; conversely, SIRT3 overexpression suppresses HIF-1α stability and activity, thereby influencing metabolic alterations (Lee et al., 2017). Thus, metabolic reprogramming serves as a core biological process linking tumorigenesis, immune evasion, pathogen infection, and therapeutic responses, making a deep understanding of its mechanisms essential for developing novel treatment strategies.

This review conducted a comprehensive literature search using the PubMed database to screen relevant studies. The search strategy employed the following keywords: “*Helicobacter pylori/H. pylori*,” “metabolic reprogramming,” “lipid metabolism,” “glucose metabolism,” “lactate metabolism,” “amino acid metabolism,” “gastric cancer,” “virulence factors,” and related terms. Boolean operators (AND, OR) were used to appropriately combine the terms. The inclusion criteria were: (1) original research articles and review articles; (2) studies focusing on *H. pylori* infection and host metabolic changes; (3) studies published in English. Exclusion criteria included: (1) studies not directly related to Helicobacter pylori or metabolism; (2) conference abstracts.

**Figure 1:**
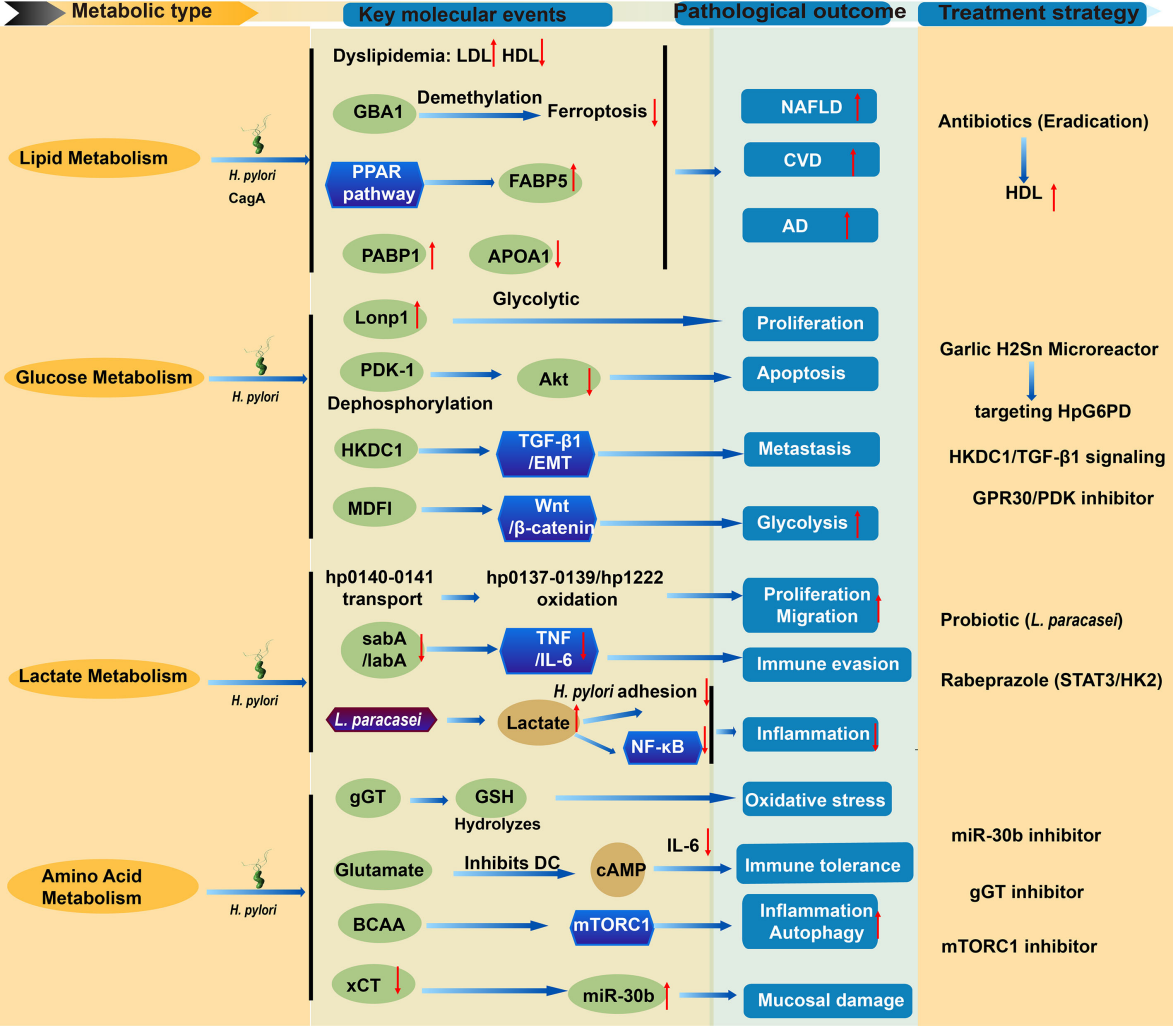
Pathophysiological mechanisms of metabolic reprogramming induced by *H. pylori* infection and potential therapeutic interventions. The diagram illustrates how *H. pylori* virulence factors (CagA^+^, gGT, lactate utilization genes *hp0137–0141/hp1222*) disrupt host metabolic pathways, driving disease progression. Lipid metabolism dysregulation: CagA^+^ strains inhibit PPAR signaling and fatty acid degradation, upregulate FABP5, and alter lipid marker expression (FABP1↑, APOA1↓). Epigenetic demethylation activates GBA1, suppressing ferroptosis and promoting atherogenic dyslipidemia (↑LDL, ↓HDL, ↑TG), which contributes to hepatic steatosis (NAFLD), cardiovascular disease (CVD), and Alzheimer’s disease (AD). Glucose metabolism reprogramming: Infection induces Lonp1-mediated glycolytic switching and PDK1 dephosphorylation, destabilizing Akt signaling and disrupting the apoptosis/proliferation balance. HKDC1 drives EMT via TGF-β1, while MDFI activates Wnt/β-catenin signaling—collectively promoting gastric carcinogenesis, proliferation, and metastasis. lactate metabolism: *H. pylori* imports host-derived lactate via *hp0140–0141* and metabolizes it through *hp0137–0139/hp1222*. Lactate serves as a chemoattractant, enhancing bacterial proliferation and migration. Concurrently, lactate uptake inhibits complement activation, downregulates adhesins (*sabA*/*labA*), and suppresses TNF/IL-6 secretion in macrophages, facilitating immune evasion and gland colonization. Amino acid metabolism disruption: gGT hydrolyzes GSH into glutamate, depleting antioxidants and inducing oxidative stress. Glutamate inhibits dendritic cell cAMP signaling and IL-6 production, promoting immune tolerance. BCAA accumulation activates mTORC1, modulating inflammation and autophagy to support bacterial colonization. xCT inhibition upregulates miR-30b, exacerbating mucosal damage. Disease outcomes: Metabolic dysregulation drives gastric pathologies (cancer, mucosal damage), systemic inflammation, NAFLD, CVD, and AD. Therapeutic strategies: Eradication therapy (antibiotics) improves lipid profiles; garlic H_2_S_n_-loaded microreactors target HpG6PD; rabeprazole inhibits STAT3/HK2 signaling; targeting of HKDC1/TGF-β1 signaling; GPR30/PDK inhibitors; probiotics (*L. paracasei*) increase lactate and block NF-κB signaling; and inhibitors of miR-30b, mTORC1, or gGT may mitigate mucosal damage and immune tolerance. CagA^+^, Cytotoxin-associated gene A positive; gGT, γ-glutamyl transpeptidase; PPAR, Peroxisome proliferator-activated receptor; FABP, Fatty acid-binding protein; GBA1, Glucocerebrosidase; LDL, Low-density lipoprotein; HDL, High-density lipoprotein; TG, Triglycerides; NAFLD, Non-alcoholic fatty liver disease; CVD, Cardiovascular disease; AD, Alzheimer’s disease; Lonp1, Lon protease 1; PDK1, Pyruvate dehydrogenase kinase 1; EMT, Epithelial–mesenchymal transition; MDFI, MyoD family inhibitor; TNF, Tumor necrosis factor; GSH, Glutathione; BCAA, Branched-chain amino acid; xCT, Cystine/glutamate antiporter; HpG6PD, *H. pylori* glucose-6-phosphate dehydrogenase; HK2, Hexokinase 2; DC, Dendritic cell.

Despite the core concept of “metabolic reprogramming” being crucial in tumorigenesis, immune evasion, and infection, a systematic understanding is lacking regarding how *H. pylori* infection triggers and regulates this process in host GECs and the microenvironment, or how it specifically drives progression from chronic inflammation to GC. This review therefore systematically integrates current research on the interaction between *H. pylori* infection and host metabolic reprogramming (**Table 1**–**4**, **Figure 1**). It aims to elucidate the pivotal role of metabolic reprogramming in *H. pylori* pathogenesis, provide new insights into disease progression, and lay the groundwork for developing novel metabolism-targeted diagnostics, preventions, and therapies for *H. pylori*-related diseases, especially GC.

**Table 1:** Impact of *H. pylori* infection on lipid metabolism: mechanisms, abnormalities, and therapeutic interventions.

Mechanism/pathway	Resulting metabolic alterations & consequence	Therapeutic intervention	References
Systemic dyslipidemia: Induces a systemic pro-atherogenic lipid profile.	Blood lipid abnormalities: Total Cholesterol (TC) ↑; Triglycerides (TG)↑; Low-Density Lipoprotein Cholesterol (LDL-C) ↑; High-Density Lipoprotein Cholesterol (HDL-C) ↓; ApoB/ApoA1 ↑. Disease risk: Accelerates atherosclerosis, increasing the risk of cardiovascular and cerebrovascular events, peripheral vascular disease, and acute coronary syndrome; Increases the risk of non-alcoholic fatty liver disease (NAFLD).	Eradication therapy: Can partially reverse dyslipidemia. Is accompanied by the downregulation of key lipid regulatory genes (e.g., FABP1, MTP). Consistently increases HDL-C and apolipoprotein A1 (ApoA1) levels.	(Niemelä et al., 1996; Buzás, 2014; Abdel-Razik et al., 2018; Fang et al., 2024; Xiao et al., 2024; Doulberis et al., 2025; Liu et al., 2025)
Interference with PPAR signaling pathway and fatty acid degradation: Infection with CagA^+^ strains leads to significant enrichment of the “PPAR signaling pathway” and “fatty acid degradation pathway”.	Hepatic lipid metabolism disorder: Exacerbates high-fat diet (HFD)-induced pathological hepatic steatosis (fatty liver). Elevates hepatic lipid metabolic parameters. Alters the expression of Fatty acid-binding protein 5 (FABP5).	Eradication therapy: Can reduce the NAFLD-Liver Fat Score (NAFLD-LFS) and Hepatic Steatosis Index (HSI). Mitigates the worsening of lipid metabolism.	(Abdel-Razik et al., 2018; Wang et al., 2022; Chen et al., 2024)
Epigenetic modification (GBA1 gene demethylation): In gastric carcinogenesis, infection triggers demethylation of the glucocerebrosidase (GBA1) gene promoter, leading to its upregulation.	Inhibition of cell death processes: Reduces lipid peroxidation levels. Increases glutathione content. Inhibits ferroptosis in gastric cancer (GC) cells, potentially promoting cancer cell survival.	–	(Shen et al., 2025)
Metabolic reprogramming of intestinal epithelial cells: Infection causes specific overexpression of key markers regulating lipid metabolism and glycolysis in intestinal epithelial cells.	Local intestinal metabolic changes: Altered lipid metabolism markers: Changes in expression of FABP1, APOC3, ANPEP, APOA1, APOA4, ALDOB, etc. Altered glycolysis markers: Changes in expression of PCK1, ADH4, etc. Closely associated with biological processes including fat digestion and absorption, the PPAR signaling pathway, and cholesterol metabolism.	–	(Li et al., 2024)
Chronic inflammation: Infection causes persistent systemic and local inflammation.	Synergistic promotion of metabolic diseases: Acts synergistically with lipid metabolism disorders to increase the risk of NAFLD, cardiovascular disease, and Alzheimer’s disease (AD).	Eradication therapy: Eliminates the source of chronic infection, thereby reducing the associated systemic inflammation.	(Buzás, 2014; Abdel-Razik et al., 2018; Doulberis et al., 2025)

## 2 Lipid metabolism

Lipogenesis refers to the process by which organisms convert excess ingested carbon sources like carbohydrates into fatty acids, which are further synthesized into triacylglycerols. Glucose, glutamine, lactate, ethanol, etc., are converted into acetyl-CoA, which is transported from the mitochondria to the cytosol via the citrate–pyruvate shuttle. Acetyl-CoA is then used in a multi-step reaction to synthesize palmitic acid (a 16-carbon saturated fatty acid). Triglycerides are assembled by esterifying a glycerol backbone (derived from 3-phosphoglycerate) stepwise to form storage fats (Song et al., 2018). Lipids are fundamental components of cell membranes and play crucial roles in mediating intracellular oncogenic signaling, endoplasmic reticulum stress, and the bidirectional crosstalk between TME cells and cancer cells (Beloribi-Djefaflia et al., 2016).

Tumor cells reprogram their lipid metabolism (increasing uptake, synthesis, oxidation, and storage) to survive hypoxic and nutrient-deficient conditions. This reprogramming, driven by genetic mutations and environmental pressures, reshapes the TME and induces lipid metabolic alterations in various TME cells, including cancer-associated fibroblasts (CAFs), regulatory T cells (Tregs), CD8^+^ T cells, and tumor-associated macrophages (TAMs) (Munir et al., 2019; Jin et al., 2023). Such metabolic adaptability (or metabolic flexibility) enables cancer cells to support rapid proliferation, sustained growth, and survival under hostile conditions (Boroughs and DeBerardinis, 2015; Obre and Rossignol, 2015; Melone et al., 2018). Therefore, therapeutic strategies targeting these adaptive metabolic pathways hold significant promise for future cancer treatment.

Notably, external factors such as chronic *H. pylori* infection also significantly contribute to systemic dyslipidemia and influence cancer-related metabolic reprogramming. *H. pylori* infection can trigger lipid metabolism disruption, characterized by elevated low-density lipoprotein (LDL) and reduced high-density lipoprotein (HDL), thereby promoting the risk of fatty liver diseases (such as non-alcoholic fatty liver disease, NAFLD), cardiovascular diseases, and Alzheimer’s disease (AD) (Doulberis et al., 2025). It is increasingly recognized as a contributor to dyslipidemia. Accumulating evidence indicates that infected individuals exhibit a characteristic atherogenic lipid profile, marked by elevated levels of total cholesterol (TC), triglycerides (TG), and low-density lipoprotein cholesterol (LDL-C), along with reduced high-density lipoprotein cholesterol (HDL-C) (Niemelä et al., 1996; Abdel-Razik et al., 2018; Xiao et al., 2024). This pattern was initially observed in early Finnish studies and subsequently confirmed by a large-scale Chinese investigation, which further identified an elevated ApoB/ApoA1 ratio as an independent risk factor for the infection (Liu et al., 2025). The resulting pro-atherogenic state is believed to accelerate the development of atherosclerosis, thereby increasing the risk of cardiovascular and cerebrovascular events, as well as peripheral vascular disease (Buzás, 2014). Additionally, through mechanisms involving chronic inflammation and altered lipid metabolism, *H. pylori* infection may elevate the risk of NAFLD (Abdel-Razik et al., 2018).

Importantly, eradication therapy has been shown to partially reverse these metabolic abnormalities, accompanied by downregulation of key lipid regulatory genes such as FABP1 and MTP, and a consistent increase in HDL-C and apolipoprotein A levels (Tsai et al., 2006; Buzás, 2014; Iwai et al., 2019; Watanabe et al., 2021). Furthermore, *H. pylori* eradication can reduce NAFLD-Liver Fat Score (NAFLD-LFS) and Hepatic Steatosis Index (HSI) (Abdel-Razik et al., 2018), and mitigate the worsening of lipid metabolism (Wang et al., 2022). Notably, one study focusing on the Chinese population yielded contrasting findings. It reported that *H. pylori* infection itself did not significantly affect lipid metabolism markers in this population but was associated with a 4-fold increased risk of acute coronary syndrome. This heightened risk was independent of *H. pylori* virulence factors such as CagA or vacuolating cytotoxin A (VacA) (Fang et al., 2024). Overall, existing evidence suggests that *H. pylori* infection, potentially through disrupting lipid metabolism (particularly elevating LDL and lowering HDL) and inducing inflammation, acts as a significant contributing factor in promoting the pathogenesis of fatty liver disease, cardiovascular disease, and AD. Eradicating *H. pylori* infection offers a beneficial strategy for improving lipid metabolism abnormalities and reducing the associated disease risks.

At the mechanistic level, *H. pylori* infection disrupts host lipid metabolism and cell death processes through multiple pathways. Specifically, infection with CagA^+^ strains of *H. pylori* was found to exacerbate high-fat diet (HFD)-induced pathological hepatic steatosis and elevate lipid metabolic parameters. RNA-seq analysis revealed that in the HFD group, differentially expressed genes were significantly enriched in the “fatty acid degradation pathway” and the “PPAR signaling pathway,” with fatty acid-binding protein 5 (FABP5) showing differential expression specifically in the context of CagA^+^ *H. pylori* infection (Chen et al., 2024). In the context of gastric carcinogenesis, *H. pylori* infection triggers demethylation of the glucocerebrosidase (GBA1) gene promoter, leading to its upregulation. This subsequently reduces lipid peroxidation levels and increases glutathione content, ultimately inhibiting ferroptosis in GC cells (Shen et al., 2025). Furthermore, within the intestinal epithelial cell population, key markers regulating lipid metabolism (such as FABP1, APOC3, ANPEP, APOA1, APOA4, and ALDOB) and markers regulating glycolysis (such as PCK1 and ADH4) were found to be specifically overexpressed. Further enrichment analysis indicated that these changes are closely associated with biological processes including fat digestion and absorption, the PPAR signaling pathway, and cholesterol metabolism (Li et al., 2024). Collectively, these findings demonstrate that *H. pylori* infection—particularly by CagA^+^ strains—may promote lipid metabolic dysregulation by interfering with core pathways such as PPAR signaling, inducing epigenetic modifications (e.g., demethylation of GBA1), and altering the expression of key metabolic markers in intestinal epithelial cells (**Table 1**, **Figure 1**).

## 3 Glucose metabolism

Glucose metabolism involves the breakdown, transformation, and utilization of glucose to provide energy (ATP) and biosynthetic precursors for life processes. Multiple metabolic pathways participate in this process. Glycolysis occurs in the cytoplasm, converting glucose to pyruvate; the most critical step is the phosphorylation of glucose to glucose-6-phosphate (G6P), which serves as a convergence point for glycolysis, the pentose phosphate pathway (PPP), the hexosamine pathway, and glycogen synthesis. Rate-limiting enzymes in this process include hexokinase (HK), phosphofructokinase-1 (PFK-1), and pyruvate kinase (PK). Under aerobic conditions, pyruvate is converted to acetyl-CoA for entry into the tricarboxylic acid (TCA) cycle; under anaerobic conditions, it is converted to lactate, thereby regenerating NAD^+^ to sustain glycolysis. The TCA cycle takes place in the mitochondrial matrix, utilizing acetyl-CoA derived from glucose, fatty acids, and amino acids. Key enzymes in this cycle are citrate synthase, isocitrate dehydrogenase (the rate-limiting enzyme), and α-ketoglutarate dehydrogenase. The PPP occurs in the cytoplasm, where glucose-6-phosphate dehydrogenase (G6PD) catalyzes the conversion of G6P to ribose-5-phosphate, a precursor for nucleotide/nucleic acid synthesis (Hay, 2016; Li and Zhang, 2016).

Beyond its role in cellular energy supply, glucose metabolic reprogramming is also implicated in various pathological processes. For instance, *H. pylori* infection has been shown to exacerbate glucose metabolism abnormalities and promote atherosclerosis (Yoshikawa et al., 2007; Vijayvergiya and Vadivelu, 2015). Specifically, *H. pylori* infection synergizes with a HFD to worsen gut microbiota dysbiosis (loss of diversity, increased Helicobacter/reduced Lactobacillus), significantly impairing the ability of antibiotics to repair HFD-induced glucose metabolism abnormalities (hyperglycemia, insulin resistance), and causing persistent Klebsiella-dominated dysbiosis (Peng et al., 2021). Thus, *H. pylori* infection not only disrupts glucose metabolism but also alters gut flora, thereby hindering the efficacy of antibiotic treatment in diet-induced metabolic disorders.

At the molecular level, *H. pylori* infection contributes to gastric carcinogenesis through multiple mechanisms. In *H. pylori*-infected GECs, the significantly induced Lon protease (Lonp1) promotes excessive cell growth by regulating mitochondrial stress response and driving a glycolytic metabolic switch, thereby driving gastric carcinogenesis (Luo et al., 2016). *H. pylori* infection induces dephosphorylation of PDK1 (at serine 241) in GECs, leading to abnormal Akt phosphorylation and degradation, disrupting cell survival signaling, and ultimately breaking the dynamic balance between apoptosis and proliferation (King and Obonyo, 2015). In CagA-positive GC cells, targeted inhibition of Akt phosphorylation or key glycolytic enzymes (HK2/LDHA) synergistically enhances 5-Fu’s killing effect on CagA-overexpressing cells, significantly reversing their drug resistance (Gao et al., 2020). In *H. pylori*-induced GC progression, significantly elevated HKDC1 drives epithelial-mesenchymal transition (EMT) via the TGF-β1/p-Smad2 signaling axis, promoting proliferation and metastasis; inhibiting HKDC1 or TGF-β1 effectively reverses this (Fang et al., 2024). Knocking down MyoD family inhibitor (MDFI) suppresses *H. pylori*-induced GC cell proliferation and enhanced glycolysis; the infection promotes MDFI expression and activates the Wnt/β-catenin pathway (Mi et al., 2022). Collectively, *H. pylori* may drive carcinogenesis via metabolic reprogramming (e.g., Lonp1-mediated glycolytic switch), disrupted signaling (PDK1/Akt), and facilitation of EMT (e.g., HKDC1/TGF-β1, MDFI/Wnt pathways).

*H. pylori* infection also modulates immunometabolism and inflammation. In *H. pylori*-induced pediatric gastritis, Src homology region 2 domain-containing phosphatase-2 (SHP2) promotes inflammation by activating macrophage glycolysis, with its downstream transcription factor SPI1 regulating metabolic gene expression and inflammation (Li et al., 2022). Bromodomain-containing protein 4 (BRD4) mediates *H. pylori* clearance by macrophages through HIF-1α-mediated transcriptional activation of glycolytic genes (Slc2a1 and Hk2) and stabilizing Nos2 mRNA to promote NO production; its loss impairs glycolysis, reduces NO bactericidal capacity, lessens inflammation but increases bacterial colonization *in vivo* (Modi et al., 2024). Thus, *H. pylori* manipulates immune cell metabolism—promoting inflammation via SHP2/glycolysis and influencing bacterial clearance via BRD4-mediated glycolytic activation and NO production.

Several potential therapeutic strategies targeting metabolic pathways have been proposed. *H. pylori* glucose-6-phosphate dehydrogenase (HpG6PD) may be a potential target for novel anti-*H. pylori* drugs (Ortiz-Ramírez et al., 2022). Hydrogen polysulfides (H_2_S_n_) from garlic inhibit *H. pylori* metabolism by targeting and inactivating HpG6PDH; converting organic sulfur to Fe_3_S_4_ greatly boosts H_2_S_n_ yield, which can be encapsulated into gastric-adapted microreactors (GAPSR), enabling highly efficient (250-fold increase), rapid, and microbiota-friendly *H. pylori* eradication in a single dose (Wang et al., 2025). Rabeprazole inhibits GC cell proliferation by suppressing STAT3 phosphorylation and its binding to the HK2 promoter, thereby reducing HK2-mediated glycolysis—an effect reversible by exogenous STAT3 overexpression (Zhou et al., 2021). GPR30 (a G-protein-coupled form of the estrogen receptor) is specifically expressed in chief cells. Expression of mutant Kras in these cells or administration of a high dose of *H. pylori* infection both lead to a reduction in the number of labeled chief cells. Upon metaplastic stimulation, chief cells are eliminated from the epithelium via a cell competition mechanism dependent on GPR30 and pyruvate dehydrogenase kinase (PDK) activity; conversely, loss of GPR30 or inhibition of PDK activity effectively preserves the chief cell population (Hata et al., 2020). In summary, these strategies—targeting *H. pylori* metabolism (inhibition of HpG6PDH, garlic-derived H_2_S_n_ microreactors) and host pathways (STAT3/HK2, GPR30/PDK)—have shown efficacy in experimental models through unique mechanisms. Their effectiveness highlights the potential of dual-targeting therapeutic strategies, though further clinical studies are needed for validation. (**Table 2**, **Figure 1**)

**Table 2:** Impact of *H. pylori* infection on glucose metabolism: mechanisms, abnormalities, and therapeutic interventions.

Mechanism/pathway	Resulting metabolic alterations & consequence	Therapeutic intervention	References
Exacerbation of systemic glucose metabolism abnormalities: Synergizes with a high-fat diet (HFD) to worsen gut microbiota dysbiosis (loss of diversity, increased Helicobacter/reduced Lactobacillus).	Systemic metabolic abnormalities: Worsens hyperglycemia and insulin resistance. Impairs the ability of antibiotics to repair HFD-induced glucose metabolism abnormalities. Causes persistent Klebsiella-dominated dysbiosis. Disease Risk: Promotes atherosclerosis.	Eradication therapy may address the root cause.	(Yoshikawa et al., 2007; Vijayvergiya and Vadivelu, 2015; Peng et al., 2021)
Driving gastric carcinogenesis (via metabolic reprogramming & signaling disruption): Upregulated Lon protease (Lonp1) promotes growth via aberrant Akt and Wnt/β-catenin signaling, enhancing glycolysis and proliferation.	Cellular phenotypic changes: Drives gastric carcinogenesis. Disrupts the dynamic balance between apoptosis and proliferation. Promotes gastric cancer cell proliferation, glycolysis, and drug resistance.	Targeted inhibition of Akt phosphorylation or key glycolytic enzymes (HK2/LDHA) synergistically enhances the killing effect of 5-Fu and reverses drug resistance.	(King and Obonyo, 2015; Luo et al., 2016; Gao et al., 2020; Mi et al., 2022)
Promoting epithelial-mesenchymal transition (EMT) and Metastasis: HKDC1 is significantly elevated, driving EMT via the TGF-β1/p-Smad2 signaling axis.	Cancer Progression: Promotes gastric cancer cell proliferation and metastasis.	Inhibiting HKDC1 or TGF-β1 effectively reverses EMT and the metastatic phenotype.	(Fang et al., 2024)
Modulating immunometabolism: SHP2 promotes inflammation by activating macrophage glycolysis. BRD4 mediates bacterial clearance through HIF-1α-mediated transcriptional activation of glycolytic genes (Slc2a1, Hk2) and stabilizing Nos2 mRNA to promote NO production.	Immune consequences: The SHP2/glycolysis axis promotes inflammatory responses. Loss of BRD4 impairs glycolysis, reduces NO bactericidal capacity, lessens inflammation but increases bacterial colonization *in vivo*.	This study focuses on mechanistic insights	(Li et al., 2022; Modi et al., 2024)
Therapy targeting pathogen metabolism: Targeting *H. pylori* glucose-6-phosphate dehydrogenase (HpG6PD).	Antibacterial Effect: HpG6PD is a potential target for novel anti-*H. pylori* drugs.	Garlic-derived H_2_S_n_ targets and inactivates HpG6PD. Conversion to Fe_3_S_4_ and encapsulation into GAPSR microreactors enables efficient, rapid, and microbiota-friendly single-dose eradication.	(Ortiz-Ramírez et al., 2022; Wang et al., 2025)
Therapy targeting host metabolic pathways: Targeting host glycolytic signaling pathways.	Anti-cancereffect: Inhibits gastric cancer cell proliferation and glycolysis.	Rabeprazole suppresses proliferation by inhibiting STAT3 phosphorylation and its binding to the HK2 promoter, reducing HK2-mediated glycolysis. Inhibiting GPR30 or PDK prevents the loss of chief cells during metaplastic stimulation.	(Hata et al., 2020; Zhou et al., 2021)

## 4 Lactate metabolism

Lactate metabolism is a dynamic process involving multi-tissue coordination, centered on the recycling of lactate between hypoxic and oxygen-rich tissues through the “lactate shuttle.” Most glucose is converted to pyruvate via glycolysis, where lactate dehydrogenase (LDH) reduces pyruvate to lactate in an NADH-dependent manner. This regenerates NAD^+^ to sustain ongoing glycolysis, particularly under hypoxia, while simultaneously clearing intracellular protons (H^+^) to mitigate acidosis, as observed in the TME. Lactate and H^+^ are co-transported out of the cell via monocarboxylate transporters (MCT1 and MCT4), maintaining intracellular pH homeostasis and acidifying the extracellular space. During aerobic metabolism, lactate is oxidized back to pyruvate by LDH. Pyruvate is then converted to acetyl-CoA by pyruvate dehydrogenase (PDH) for entry into the TCA cycle, generating ATP for energy production. During starvation or post-exercise, lactate is transported to the liver via the Cori cycle to generate glucose (Ippolito et al., 2019; Brooks, 2020; Li et al., 2022). Additionally, lactate serves as a metabolic precursor for fatty acid synthesis and amino acid biosynthesis.

Notably, the gastric pathogen *H. pylori* has evolved sophisticated mechanisms to utilize lactate, enhancing its survival and virulence. *H. pylori* utilizes lactate through the synergistic action of three gene clusters: *hp0137–0139* (L-LDH), *hp1222* (D-LDH), and *hp0140–0141* (lactate transporter). Specifically, *hp0140–0141* mediates the transport of both D-lactate and L-lactate; *hp0137–0139* mediates the NAD-dependent oxidation of L-lactate; and *hp1222* mediates the NAD-independent oxidation of D/L-lactate (Iwatani et al., 2014). In a gastric organoid co-culture system, L-lactate secreted by gastric mucosal cells significantly promotes *H. pylori* proliferation (Takahashi et al., 2007). Furthermore, L-lactate serves as an important chemoattractant guiding bacterial migration (Machuca et al., 2017). Critically, through uptake of host-derived L-lactate, *H. pylori* disrupts the stability of surface C4b binding, specifically inhibiting activation of the classical complement pathway. This enables the bacterium to resist lytic effects and promotes its colonization within the gastric glands, particularly in the corpus region, identifying lactate as a key factor conferring complement resistance (Hu and Ottemann, 2023).

Both L- and D-lactate can downregulate the expression of the sabA and labA genes, though this process is independent of the lactate chemosensor TlpC. The downregulation of sabA is mediated by the ArsRS two-component system, while *labA* is regulated through the CheA/CheY system, indicating distinct molecular mechanisms underlying their response to lactate. Additionally, lactate can inhibit *H. pylori*-induced production of pro-inflammatory cytokines in macrophages without affecting their phagocytic ability. Notably, SabA, a key virulence factor, mediates bacterial adhesion to host cells during inflammation (Somiah et al., 2022). In a related probiotic context, *Lactobacillus paracasei* 06TCa19 significantly increases lactate concentration, simultaneously suppressing *H. pylori* adhesion and CagA protein injection while blocking the NF-κB/p38 MAPK inflammatory signaling pathway, thereby comprehensively downregulating IL-8/RANTES gene transcription and protein synthesis (Takeda et al., 2017).

Beyond its role in bacterial pathogenesis, lactate also serves as a key immunomodulatory metabolite in the TME. It contributes to an immunosuppressive milieu by inhibiting the function of effector immune cells, promoting the differentiation of immunosuppressive cells, and participating in epigenetic modifications (Heuser et al., 2023). Its effects are both cell-type and context-dependent, establishing lactate as a critical metabolic checkpoint that links tumor metabolism with immune evasion (Heuser et al., 2023). For instance, lactate can skew macrophages toward an M2-like polarized state (Zhang and Li, 2020). Lactylation, a novel histone modification induced by lactate, has been shown to play a dual role in macrophage-mediated immune responses: it generally promotes anti-inflammatory and repair phenotypes, though in certain gastrointestinal contexts it may instead accelerate tumor progression and induce immunosuppression (Che et al., 2025). Additionally, lactate recruits regulatory T (Treg) cells via the G-protein-coupled receptor 81 (GPR81) signaling axis to foster immune tolerance. Deficiency in this pathway releases the suppression of CD8^+^ T cells, thereby inhibiting GC progression (Su et al., 2024). In highly glycolytic tumor microenvironments, Treg cells actively take up lactate through monocarboxylate transporter 1 (MCT1), which promotes nuclear translocation of NFAT1 and subsequent enhancement of PD-1 expression. Conversely, PD-1 expression in effector T cells is suppressed under the same conditions (Kumagai et al., 2022).

Most lactate-targeting strategies remain preclinical, focusing on inhibiting *H. pylori* lactate uptake/metabolism or disrupting lactate-mediated immunosuppression in the TME (Heuser et al., 2023; Gu et al., 2025). However, as lactate is a central metabolic and signaling molecule, systemic targeting poses risks—including disrupted energy homeostasis, acidosis, or unintended immunomodulation. Future translation requires developing tissue-specific delivery systems (e.g., gastric-targeted nanoparticles) and combination strategies that selectively inhibit microbial or tumor-related lactate pathways without altering systemic physiological functions.

In summary, these findings reveal that *H. pylori* coordinately utilizes host-derived lactate through multiple synergistic pathways—stimulating its own growth, guiding migration, and critically disabling host immune defenses by suppressing complement-mediated lysis and pro-inflammatory signaling. This multifaceted lactate-dependent strategy facilitates its successful colonization within the protected niche of the gastric glands. Furthermore, lactate’s role as an immunometabolite in the TME adds another layer of complexity to its involvement in *H. pylori*-associated gastric pathogenesis and carcinogenesis (**Table 3**, **Figure 1**).

**Table 3:** Interaction between *H. pylori* and lactate metabolism: mechanisms, abnormalities, and interventions.

Mechanism/pathway	Resulting metabolic alterations & consequence	Therapeutic intervention	References
*H. pylori* utilization of lactate & Immune evasion: Utilizes host D/L-lactate via three synergistic gene clusters: hp0140-0141(transporter); hp0137-0139(L-LDH); hp1222(D-LDH).	Enhanced bacterial survival & Virulence: • Promotes Proliferation: L-lactate secreted by gastric mucosal cells significantly promotes *H. pylori* proliferation. • Guides Migration: L-lactate acts as a chemoattractant guiding bacterial migration. • Resists Complement: Utilization of host L-lactate disrupts surface C4b binding stability, inhibiting classical complement pathway activation, enabling resistance to lysis and promoting colonization within gastric glands (particularly in the corpus).	• Probiotics: *Lactobacillus paracasei* 06TCa19 increases lactate concentration, suppressing *H. pylori* adhesion, CagA injection, and the NF-κB/p38 MAPK inflammatory pathway. • Targeting lactate utilization: Strategies to inhibit *H. pylori* lactate uptake/metabolism (preclinical stage).	(Takahashi et al., 2007; Iwatani et al., 2014; Machuca et al., 2017; Takeda et al., 2017; Hu and Ottemann, 2023)
Lactate modulation of *H. pylori* virulence genes: L/D-lactate downregulates virulence genes sabA and labA expression (independent of the TlpC sensor). sabA downregulation is mediated by the ArsRS two-component system; labA is regulated via the CheA/CheY system.	Virulence & Inflammation modulation: Downregulates SabA, a key virulence factor mediating bacterial adhesion during inflammation. Inhibits *H. pylori*-induced production of pro-inflammatory cytokines in macrophages (without affecting phagocytic ability).	Potential intervention: Using lactate analogs or signaling modulators to downregulate virulence gene expression	(Somiah et al., 2022)
Lactate’s immunosuppressive role in the TME: Acts as an immunometabolic checkpoint linking tumor metabolism to immune evasion. Promotes anti-inflammatory/repair phenotypes via histone lactylation (may accelerate tumor progression in certain GI contexts). Recruits Treg cells via the GPR81 signaling axis to foster immune tolerance. Treg cells uptake lactate via MCT1, promoting NFAT1 nuclear translocation to enhance PD-1 expression.	Remodeling of tumor immune microenvironment: Inhibits effector immune cell function and promotes immunosuppressive cell differentiation. Skews macrophages toward an M2-like polarization. Recruits Treg cells, suppressing CD8^+^ T cell function, promoting immune tolerance and gastric cancer progression. Differentially regulates PD-1 expression in Tregs vs. effector T cells.	Most targeting strategies are in the preclinical stage, aiming to disrupt lactate-mediated immunosuppression (e.g., inhibiting MCTs, targeting GPR81 or lactylation). Challenge: Requires developing tissue-specific delivery systems (e.g., gastric-targeted nanoparticles) and combination strategies to avoid disrupting systemic energy homeostasis.	(Zhang and Li, 2020; Heuser et al., 2023)

## 5 Amino acid metabolism

Amino acids are crucial organic molecules containing amino and carboxyl groups, categorized as α-, β-, γ-, or δ- amino acids based on the position of these functional groups. Among these, the 22 α-amino acids that constitute proteins are the most significant. They serve not only as fundamental building blocks for synthesizing proteins, polypeptides, and other biomolecules, but also as an energy source via oxidative pathways involving deamination and the urea cycle (Brosnan, 2000). Cellular uptake of amino acids relies on amino acid transporters (AATs). These transporters not only facilitate the transmembrane transport of amino acids but also act as sensors of amino acid concentration and initiators of nutrient signaling, and can be classified based on their substrates and mechanisms (Hediger et al., 2013). Beyond their core roles as biosynthetic precursors and energy sources, amino acids profoundly regulate key cellular processes: They activate signaling pathways like mTOR (via glutamine, arginine, leucine, etc.) to regulate protein synthesis and cell growth; modulate metabolic pathways (such as gluconeogenesis and the urea cycle) through specific amino acids (e.g., alanine, arginine); and play indispensable roles in the proliferation, activation, and function of immune cells (e.g., T cells). Deficiency in certain amino acids (e.g., tryptophan, arginine, leucine) directly impairs immune cell function (Paulusma et al., 2022; Ling et al., 2023; Yang et al., 2023; Chen et al., 2024). Therefore, amino acids are essential, multifunctional molecules vital for sustaining life activities, integral to core biological processes including biosynthesis, energy metabolism, signal transduction, and immune regulation.

In the context of infection, *H. pylori* extensively disrupts host amino acid metabolism to facilitate its survival and persistence. Metabolomic analyses, such as nuclear magnetic resonance (NMR), reveal that *H. pylori* infection induces systemic metabolic alterations, including abnormalities in phenylalanine/tyrosine metabolism, pterin biosynthesis, starch/sucrose metabolism, and galactose metabolism pathways (Fulgione et al., 2020). To adapt to and manipulate the gastric environment, *H. pylori* employs a dual enzyme-mediated mechanism: Its biotin protein ligase (HpBPL) activates fatty acid synthesis, gluconeogenesis, and amino acid catabolism, enhancing bacterial survival in the acidic gastric environment (Ayanlade et al., 2025). Meanwhile, γ-glutamyl transpeptidase (gGT) hydrolyzes glutathione (GSH) to glutamate and cysteinylglycine (Cys-Gly) for internalization, leading to the depletion of host antioxidant defenses due to GSH loss (Baskerville et al., 2023). Moreover, gGT-derived glutamate inhibits the dendritic cell cAMP signaling pathway, reduces IL-6 secretion, and induces immune tolerance to sustain chronic infection (Käbisch et al., 2016).

This metabolic disorder further triggers a cascade of pathological effects: *H. pylori*-induced accumulation of branched-chain amino acids (BCAAs) activates the mTORC1 signaling pathway, modulating inflammatory responses and inducing autophagy to promote bacterial colonization of the gastric mucosa (Cuomo et al., 2020). Concurrently, infection-induced inhibition of the cystine-glutamate transporter (xCT) reduces glutamate release, exacerbating gastric mucosal damage through upregulation of miR-30b, a process reversible by miR-30b inhibitors (Du et al., 2020). In summary, *H. pylori* establishes chronic infection and induces tissue damage through a self-reinforcing pathogenic cycle, which integrates metabolic reprogramming (via HpBPL and gGT), immune tolerance (gGT-mediated dendritic cell suppression), and miR-30b-dependent mucosal injury (through xCT inhibition). Targeting any node within this loop—such as modulating metabolic enzymes, inhibiting miR-30b, or restoring antioxidant and immune signaling—may offer novel opportunities for disrupting chronic infection and mitigating gastric pathology (**Table 4**, **Figure 1**).

**Table 4:** Impact of *H. pylori* infection on amino acid metabolism: mechanisms, abnormalities, and interventions.

Mechanism/pathway	Resulting metabolic alterations & consequence	Therapeutic intervention	References
Systemic metabolic reprogramming: Infection induces systemic metabolic alterations.	Metabolic pathway abnormalities: • Disorders in phenylalanine/tyrosine metabolism • Disorders in pterin biosynthesis • Disorders in starch/sucrose metabolism • Disorders in galactose metabolism	Eradication therapy may fundamentally reverse the abnormalities.	(Fulgione et al., 2020)
Dual enzyme-mediated survival & Immune evasion: • Biotin protein ligase (HpBPL): Activates fatty acid synthesis, gluconeogenesis, and amino acid catabolism, enhancing bacterial survival in the acidic gastric environment. • γ-glutamyl transpeptidase (gGT): Hydrolyzes glutathione (GSH) into glutamate and cysteinylglycine (Cys-Gly) for bacterial utilization.	Host defense mechanisms disruption: • Depletion of the host antioxidant GSH. • gGT-derived glutamate inhibits the dendritic cell cAMP signaling pathway, reduces IL-6 secretion, and induces immune tolerance to sustain chronic infection.	Potential strategy: Targeting metabolic enzymes like HpBPL or gGT may impair bacterial adaptation and virulence	(Käbisch et al., 2016; Baskerville et al., 2023; Ayanlade et al., 2025)
Activation of the mTORC1 signaling pathway: Infection induces the accumulation of branched-chain amino acids (BCAAs).	Promotion of bacterial colonization: • BCAAs activate the mTORC1 signaling pathway. •Modulates inflammatory responses and induces autophagy, thereby promoting bacterial colonization of the gastric mucosa.	Potential intervention: Using mTORC1 signaling inhibitors	(Cuomo et al., 2020)
Inhibition of the cystine-glutamate transporter (xCT): • Infection inhibits xCT function.	Exacerbation of gastric mucosal damage: • Reduces glutamate release. •Exacerbates gastric mucosal damage by upregulating miR-30b.	miR-30b inhibitors can reverse this process and alleviate mucosal damage.	(Du et al., 2020)

## 6 Conclusion and future directions

This review underscores metabolic reprogramming as a central mechanism by which *H. pylori* infection drives the progression from chronic inflammation to GC. The pathogen disrupts host lipid, glucose, lactate, and amino acid metabolism through virulence factors like CagA, leading to significant pathophysiological consequences. Key disruptions include elevated LDL and reduced HDL promoting extra-gastric diseases, enhanced glycolysis via pathways involving HKDC1, Lonp1, and PDK1/Akt fueling epithelial proliferation and EMT, exploitation of host lactate (mediated by genes like hp0140-0141) facilitating bacterial colonization and immune evasion, and amino acid imbalances (BCAA accumulation activating mTORC1, gGT depleting GSH) causing oxidative stress and immune tolerance. These metabolic alterations are fundamental to *H. pylori* pathogenesis and carcinogenesis.

The infection-induced metabolic perturbations reshape the TME, creating conditions conducive to persistence and malignancy. Metabolite accumulation (lactate) and nutrient competition suppress CD8^+^ T cell function and promote immunosuppressive phenotypes in macrophages and Tregs. Furthermore, gut microbiota dysbiosis, characterized by increased *Helicobacter* and reduced *Lactobacillus*, synergizes with metabolic dysregulation (like glucose imbalance) to form a vicious cycle that undermines antibiotic efficacy. Strategies like specific probiotics (*L. paracasei*) that increase lactate or innovative approaches such as H_2_S_n_-loaded microreactors offer novel preventive and therapeutic avenues by targeting bacterial metabolism and virulence.

Future research must prioritize several key areas to translate these insights into clinical impact. Mechanistically, deeper exploration of the spatiotemporal dynamics of strain-specific metabolic interference and how reprogramming initiates the “inflammation-metaplasia-carcinoma” cascade is essential, including dissecting crosstalk between mitochondrial stress (Lonp1), epigenetics (GBA1 methylation), and transcriptional regulation of metabolic enzymes. Technologically, applying spatial multi-omics and developing advanced organoid-microbe co-culture models are needed to resolve *in situ* host-bacteria-immune metabolic interactions within the gastric niche. Translationally, efforts should focus on screening metabolic biomarkers for early diagnosis and prognosis, designing precision drug delivery systems targeting metabolism (potentially combined with immunotherapy or antibiotics), and exploring combined “metabolic intervention-microbiota regulation” strategies for prevention. Finally, understanding host heterogeneity, such as links between genetic background (paradoxical lipid profiles in Asians), environmental factors, and metabolic phenotypes, is crucial for personalized approaches. A comprehensive understanding of *H. pylori*-driven metabolic reprogramming will pave the way for novel “metabolism-targeted therapies” aimed at blocking gastric carcinogenesis.
